# Approximate Bayesian estimation of coevolutionary arms races

**DOI:** 10.1371/journal.pcbi.1006988

**Published:** 2019-04-15

**Authors:** Scott L. Nuismer, Bob Week

**Affiliations:** 1 Department of Biological Sciences, University of Idaho, Moscow, Idaho, United States of America; 2 Bioinformatics and Computational Biology, University of Idaho, Moscow, Idaho, United States of America; University of Canterbury, NEW ZEALAND

## Abstract

Exaggerated traits involved in species interactions have long captivated the imagination of evolutionary biologists and inspired the durable metaphor of the coevolutionary arms race. Despite decades of research, however, we have only a handful of examples where reciprocal coevolutionary change has been rigorously established as the cause of trait exaggeration. Support for a coevolutionary mechanism remains elusive because we lack generally applicable tools for quantifying the intensity of coevolutionary selection. Here we develop an approximate Bayesian computation (ABC) approach for estimating the intensity of coevolutionary selection using population mean phenotypes of traits mediating interspecific interactions. Our approach relaxes important assumptions of a previous maximum likelihood approach by allowing gene flow among populations, variable abiotic environments, and strong coevolutionary selection. Using simulated data, we show that our ABC method accurately infers the strength of coevolutionary selection if reliable estimates are available for key background parameters and ten or more populations are sampled. Applying our approach to the putative arms race between the plant *Camellia japonica* and its seed predatory weevil, *Curculio camelliae*, provides support for a coevolutionary hypothesis but fails to preclude the possibility of unilateral evolution. Comparing independently estimated selection gradients acting on Camellia pericarp thickness with values simulated by our model reveals a correlation between predicted and observed selection gradients of 0.941. The strong agreement between predicted and observed selection gradients validates our method.

## Author summary

Exaggerated traits involved in species interactions, such as extreme running speeds in predator and prey, have long captivated the imagination of evolutionary biologists and inspired the durable metaphor of the coevolutionary arms race. Despite decades of research, however, we have only a handful of examples where coevolution has been rigorously established as the cause of trait exaggeration. The reason support for a coevolutionary mechanism remains elusive is that we lack generally applicable tools for quantifying the intensity of coevolution. Here we develop a computational approach for estimating the intensity of coevolutionary selection (ABC Coevolution) and illustrate its use by applying the method to a well-studied interaction between the plant *Camellia japonica* and its seed predatory weevil, *Curculio camelliae*. Our results provide support for a coevolutionary hypothesis but fail to preclude the possibility of unilateral evolution.

## Introduction

Few metaphors have captured the interest of evolutionary biologists and ecologists more than the coevolutionary arms race [1]. Whether between species, sexes, individuals, or genes, the idea of perpetually and reciprocally escalating defenses and counter-defenses has inspired an enormous amount of research [e.g., 2, 3–21]. As a result, we now have convincing evidence that arms races occur both within and between species, at least in some well-studied cases. What we know with much less certainty, however, is just how reciprocal, common, and intense evolutionary arms races tend to be across the diversity of life as a whole.

Our overall understanding of evolutionary arms races is limited by existing approaches that are labor intensive and that generally yield qualitative rather than quantitative estimates for the strength of reciprocal selection. For instance, studies exploring arms races at the level of genes often rely on classical population genetic tools that identify signatures of positive selection using ratios of synonymous to non-synonymous substitutions, patterns of linkage disequilibrium, or shifts in the site frequency spectra [22, 23]. Although the results of such studies can be consistent with a coevolutionary arms race (e.g., positive selection acting on putatively interacting host and pathogen genes), the degree of reciprocity between the species cannot be easily ascertained and alternative explanations for parallel positive selection are often plausible. Similar issues plague studies investigating arms races at the phenotypic level. Such studies often rely on fossil times series [24–26], the phylogenetic distribution of traits [13, 27, 28], or relationships between traits over space/time [2, 19, 29–32]. As with the genetic approaches, these phenotypic studies can provide evidence consistent with a coevolutionary arms race (e.g., parallel patterns of trait escalation in the fossil record, correlated traits among populations, etc.), but are generally unable to quantify the extent of reciprocity or rule out alternative explanations for parallel patterns of escalation in interacting species. Thus, we are currently in a situation where we have tools that can be used to identify parallel patterns of genetic or phenotypic escalation in interacting species pairs, but few tools that can robustly estimate the degree of reciprocal or coevolutionary selection underlying these patterns of parallel evolutionary change.

Recently, we developed a maximum likelihood approach that begins to fill this gap in the toolkit available for investigating coevolutionary arms races [33]. This approach estimates the strength of coevolutionary selection between a pair of interacting species using the spatial distribution of traits involved in the interaction. In addition to estimating the strength of coevolutionary selection, this approach opens the door to likelihood ratio tests that allow the relative support for coevolutionary and non-coevolutionary hypotheses to be evaluated. Although fast and efficient, this method relies on a handful of important assumptions. Specifically, this approach assumes interactions do not depend too strongly on the traits of the interacting individuals. In addition, the maximum likelihood approach ignores gene flow among populations and assumes random genetic drift is the only force generating phenotypic diversity among populations.

Here we develop a complementary Bayesian approach (*ABC Coevolution*) that relaxes key restrictions of the maximum likelihood framework by allowing for strong coevolutionary selection, gene flow among populations, and environmental variation in abiotic optima. Although we restrict our attention to interactions between species, the general methodology developed here could be applied to arms races between the sexes with only very minor modifications. Extending our approach to other forms of ecological interaction (e.g., mutualism) or different functional forms of interaction (e.g., trait matching) is equally straightforward. We will begin by developing a model that simulates coevolution between a pair of interacting species distributed across a landscape; these simulations will power our ABC framework. Next, we will evaluate the performance of our ABC approach using simulated data. Finally, we will apply our ABC method to a well-studied, but putative, example of a coevolutionary arms race between a seed boring weevil and its plant prey [34].

## Methods

We focus on the common scenario where the outcome of an interaction between species X and species Y depends on the mechanistic interaction between a pair of quantitative traits, *x* and *y*. For instance, in the interaction between the Japanese Camellia, *Camellia japonica*, and its seed predatory weevil, *Curculio camelliae*, the probability of seed predation depends on the size of the weevil’s rostrum relative to the thickness of the Camellia fruit’s defensive pericarp [34]. Similarly, the outcome of interaction between the newt, *Taricha granulosa*, and its garter snake predator, *Thamnophis sirtalis*, depends on the amount of tetrodotoxin produced by the newt relative to the detoxification ability of the snake [35]. The approach we develop here requires that the population mean values of these key traits, $\bar{x}$ and $\bar{y}$, be estimated in *N* different populations, as has been done in a wide range of systems [e.g., 2, 35, 36, 37–40]. Our approach then summarizes the data using five statistics: 1) the average population mean phenotype in each species over all sampled populations (*μ*_*x*_, *μ*_*y*_), 2) the standard deviation in population mean phenotypes among all sampled populations (*σ*_*x*_, *σ*_*y*_), and 3) the correlation between the population mean phenotypes of the two species over all sampled populations (*ρ*_*xy*_). With this data in hand, our approach employs approximate Bayesian computation [e.g., 41, 42–44] to develop posterior distributions for the strength of coevolution between the interacting species pair. We begin by describing the evolutionary simulations that power our ABC approach. Next, we describe how these evolutionary simulations are integrated into an approximate Bayesian framework and then describe how we evaluate the performance of the approach using simulated data. Finally, we demonstrate how our approach can be applied to real data using the well-studied interaction between the seed boring weevil, *Curculio camelliae*, and its host plant, *Camellia japonica*. All simulations and approximate Bayesian computation were conducted in C++; the source code is available at: http://www.leeef.org/resources.

### Coevolutionary simulation

We simulate coevolution between a pair of species that interact, and potentially coevolve, within the *N* spatially distributed populations for which phenotypic data has been collected. Specifically, we follow the population mean phenotypes of the two species within each of *N* populations over the course of a generation consisting of: 1) selection, 2) random genetic drift, 3) gene flow, and finally 4) random mating and inheritance. We then repeat this life cycle for one hundred generations, after which the life cycle continues until the summary statistics, *μ*_*x*_, *μ*_*y*_, *σ*_*x*_, *σ*_*y*_, and *ρ*_*xy*_ reach an approximate equilibrium where the means change by less than 1% of their values each generation and the standard deviations and correlation change by less than 5% of their values each generation, on average, over a ten generation window. Although a 5% change in standard deviations or correlation may seem inconsistent with equilibrium, this level of variation is consistent with sampling error given the relatively small number of populations we study here (i.e., < 20). In the sections that follow, we describe the details of each step of this life cycle, pointing out key assumptions along the way. All model parameters and their biological interpretations and assumptions are summarized in Table 1.

**Table 1:** Parameters, biological interpretations, and specific assumptions.

*Parameter*	*Biological interpretation*	*Relevant assumptions*
*N*	The number of populations sampled	Identical to number of populations in metapopulation
*γ*_*i*_	Strength of stabilizing selection acting on species i	Spatially homogenous
*θ*_*i*,*j*_	Phenotypic optimum favored by stabilizing selection acting on species i in population j	Spatially heterogeneous with mean *μ*_*θ*,*i*_, variance $\sigma_{\theta ,i}^{2}$, and interspecific correlation *ρ*_*θ*_
*α*_*i*_	Coevolutionary sensitivity of species i	Spatially homogenous
$h_{i}^{2}$	Heritability of key trait in species i	Spatially homogenous
*V*_*i*_	Phenotypic variance of key trait in species i	Fixed
*m*_*i*_	Rate of movement among populations in species i	Island model
*n*_*i*_	Effective population size of species i	Constant and spatially homogeneous

Natural selection–We assume that individuals of species i inhabiting population j experience stabilizing selection toward some spatially variable phenotypic optimum, *θ*_*i*,*j*_. Because correlations between abiotic optima may lead to patterns similar to those produced by coevolution [45, 46], we allow the phenotypic optima of the two species to be modestly correlated across space, with correlations ranging between -0.1 and 0.1. For simplicity, we will refer to this background selection as “abiotic” even though it may result from biotic interactions external to the focal interaction, the abiotic environment, or some combination of both. Specifically, we assume that the abiotic fitness of the two species in population j is given by:

$$W_{A,X,j}=exp\left[-\gamma_{X}{\left(x_{j}-\theta_{X,j}\right)}^{2}\right]$$ (1)

$$W_{A,Y,j}=exp\left[-\gamma_{Y}{\left(y_{j}-\theta_{Y,j}\right)}^{2}\right]$$

where *γ*_*i*_ is the strength of stabilizing selection acting on species i.

Selection imposed by the interaction between the focal species, X and Y, is assumed to depend on their relative trait values. For simplicity and brevity, we refer to this as “biotic” selection. Specifically, we assume that the fitness of an individual of species X with phenotype x in an encounter with an individual of species Y with phenotype y is given by:

$$W_{B,X,j}=\frac{1}{1+exp[-\alpha_{x}(x-y)]}$$ (2)

and the fitness of an individual of species Y with phenotype y in an encounter with an individual of species X with phenotype x is given by:

$$W_{B,Y,j}=\frac{1}{1+exp[-\alpha_{y}(y-x)]}$$

where the parameter *α*_*i*_ measures the sensitivity of the biotic component of fitness in species i within population j to the difference between the phenotypes of the individuals. These functions assume a phenotypic differences or arms race model of interaction [e.g., 47] where individual fitness is increased by having a phenotypic value that is large relative to that of the interacting individual. For selection to be reciprocal and coevolutionary, *α*_*i*_>0 for both species X and Y. Assuming encounters between individuals occur at random, Eq (2) can be used to determine the expected fitness of individuals within each species by integrating over the phenotype distribution of the interacting species:

$${\bar{W}}_{B,X,j}=\int \frac{1}{1+exp[-\alpha_{x}(x-y)]}\phi_{y,j}(y)\;dy$$ (3)

$${\bar{W}}_{B,Y,j}=\int \frac{1}{1+exp[-\alpha_{y}(y-x)]}\phi_{x,j}(x)\;dx$$

where *ϕ*_*x*,*j*_(*x*) and *ϕ*_*y*,*j*_(*y*) are the phenotype frequency distributions for traits x and y, respectively, within population j. These phenotype distributions are assumed to be normal, with means $\bar{x}$ and $\bar{y}$ and variances *V*_*X*_ and *V*_*Y*_, respectively. For simplicity, we assume the phenotypic variances, *V*_*X*_ and *V*_*Y*_, are constant over space and time.

The total lifetime fitness of individuals is assumed to be the product of the abiotic and biotic fitness components:

$$W_{T,X,j}=W_{A,X,j}{\bar{W}}_{B,X,j}$$ (4)

$$W_{T,Y,j}=W_{A,Y,j}{\bar{W}}_{B,Y,j}$$

The mean fitness of each species can then be calculated by integrating total lifetime fitness over the phenotype distribution of the focal species:

$${\bar{W}}_{T,X,j}=\int W_{T,X,j}\phi_{x,j}(x)\;dx$$ (5)

$${\bar{W}}_{T,Y,j}=\int W_{T,Y,j}\phi_{y,j}(y)\;dy$$

Total lifetime fitness (4) and population mean fitness (5) can then be used together to predict the frequency distribution of phenotypes within each species and population following selection:

$$\phi_{x,j}^{\prime }(x)=\phi_{x,j}(x)\frac{W_{T,X,j}}{{\bar{W}}_{T,X,j}}$$ (6)

$$\phi_{y,j}^{\prime }(y)=\phi_{y,j}(y)\frac{W_{T,Y,j}}{{\bar{W}}_{T,Y,j}}$$

where the primes indicate the next step in the life-cycle. The post-selection population mean phenotypes can then be calculated for each species and population by integrating the product of the trait value and post-selection phenotype frequency (6):

$${\bar{x}}_{j}^{\prime }=\int x\;\phi_{x,j}^{\prime }(x)dx$$ (7)

$${\bar{y}}_{j}^{\prime }=\int y\;\phi_{y,j}^{\prime }(y)dy$$

Random genetic drift–After selection, we assume a sample of individuals from species i equal to the local effective population size, *n*_*i*_, survives. The population mean phenotypes after this sampling process are then given by:

$${\bar{x}}_{j}^{\prime \prime }={\bar{x}}_{j}^{\prime }+\xi_{x}$$ (8)

$${\bar{y}}_{j}^{\prime \prime }={\bar{y}}_{j}^{\prime }+\xi_{y}$$

where *ξ*_*i*_ is a random variable drawn from a gaussian distribution with mean zero and variance equal to *V*_*i*_/*n*_*i*_ in species i.

Movement–We assume individuals move among populations at random, with the probability of movement between pairs of populations in species i defined by the migration matrix **M**. With this assumption, the population mean phenotype for species X in population j following movement among populations is:

$${\bar{x}}_{j}^{\prime \prime \prime }=\sum_{k=1}^{N}m_{x,j,k}{\bar{x}}_{k}^{\prime \prime }$$ (9)

and the population mean phenotype for species Y in population j following movement is:

$${\bar{y}}_{j}^{\prime \prime \prime }=\sum_{k=1}^{N}m_{y,j,k}{\bar{y}}_{k}^{\prime \prime }$$

In these expressions, *m*_*i*,*j*,*k*_ represents the entry in the migration matrix, **M**, measuring the probability an individual of species i moves from population k to population j. For the special case of the island model we focus on here where gene flow occurs at an equal rate among all populations, (9) reduces to:

$${\bar{x}}_{j}^{\prime \prime \prime }=(1-m_{x}){\bar{x}}_{j}^{\prime \prime }+\frac{m_{x}}{N-1}\sum_{\begin{array}{c} k=1\\ k\neq j \end{array}}^{N}{\bar{x}}_{k}^{\prime \prime }$$ (10)

$${\bar{y}}_{j}^{\prime \prime \prime }=(1-m_{y}){\bar{y}}_{j}^{\prime \prime }+\frac{m_{y}}{N-1}\sum_{\begin{array}{c} k=1\\ k\neq j \end{array}}^{N}{\bar{y}}_{k}^{\prime \prime }$$

where *m*_*i*_ is the proportion of individuals within each population composed of immigrants from other populations and *N* is the number of populations for which phenotypic data is available.

Random mating and inheritance–Following movement among populations, individuals mate at random and reproduce. Assuming the traits mediating the interaction are heritable, the change in the mean phenotype of species X and Y within population j is given by:

$$\Delta {\bar{x}}_{j}=h_{x}^{2}({\bar{x}}_{j}^{\prime \prime \prime }-{\bar{x}}_{j})$$ (11)

$$\Delta {\bar{y}}_{j}=h_{y}^{2}({\bar{y}}_{j}^{\prime \prime \prime }-{\bar{y}}_{j})$$

where the heritability, $h_{i}^{2}$, is assumed to be constant over both time and space.

### Approximate Bayesian Computation

Approximate Bayesian Computation is a conceptually simple rejection algorithm that implements the following steps: 1) Draw parameters of the model from prior distributions, 2) Simulate data for the selected parameters and calculate summary statistics, 3) If the summary statistics calculated from the simulated data are sufficiently close to their values in the real data, include the parameters in the posterior distribution and return to Step 1. Otherwise, do not include the parameters in the posterior distribution and return to Step 1. For well-chosen summary statistics and appropriate thresholds for acceptance into the posterior, this algorithm converges on an accurate approximation of the posterior distribution [48]. Approximate Bayesian Computation has now been applied to a wide range of problems in ecology and evolution, and its strengths and weaknesses are well-understood [41, 42, 49]. Here, we rely on previous work demonstrating that the bivariate distribution describing population mean phenotypes of coevolving species within a metapopulation can be accurately described using only five statistical moments to select our summary statistics [46]. Specifically, we summarize both simulated and real data using the average population mean phenotype of each species over the metapopulation, *μ*_*x*_ and *μ*_*y*_, the standard deviation of population mean phenotypes for each species over the metapopulation, *σ*_*x*_ and *σ*_*y*_, and the correlation between the population mean phenotypes of the two species over the metapopulation, *ρ*_*xy*_. If the values of these five summary statistics are sufficiently similar in simulated and real data, the parameters generating the simulated data are added to the posterior distribution. The result is multivariate posterior distribution for the 17 model parameters described in Tables 2 and 3. Detailed descriptions of prior distributions and thresholds for acceptance into the posterior are described in subsequent sections.

**Table 2:** Distributions for simulation parameters and prior distributions.

*Model Parameter*	*Simulation Distribution*	*Prior Distribution*
*γ*_*i*_	Gamma (0.1,10)	Uniform (0, 0.2)
*μ*_*θ*,*i*_	Gamma (5.0, 20.0)	Gamma (5.0, 20.0)
$\sigma_{\theta ,i}^{2}$	Gamma(0.01×*μ*_*θ*,*i*_, 1.5)	Gamma(0.01×*μ*_*θ*,*i*_, 1.5)
*ρ*_*θ*_	Uniform (-0.1, 0.1)	Uniform (-0.1, 0.1)
*α*_*i*_	Uniform (0.0, 2.0)^1^	Uniform (0.0, 2.0)
$h_{i}^{2}$	Uniform (0.2, 0.8)	Uniform (0.2, 0.8)
*V*_*i*_	1.0	1.0
*m*_*i*_	Gamma (0.001, 10.0)	Gamma (0.001, 10.0)
*n*_*i*_	Gamma (1000.0, 10.0)	Gamma (1000.0, 10.0)

^1^ When simulating data, *α*_*i*_ was drawn from this distribution only after first clearing a hurdle value of 0.25. Failure to clear the hurdle resulted in *α*_*i*_ being equal to zero.

**Table 3:** Distributions for simulation parameters and refined prior distributions.

*Model Parameter*	*Simulation Distribution*	*Prior Distribution*
*γ*_*i*_	Gamma (0.05, 5.0)	Gamma (*γ*_*i*_, 200.0)
*μ*_*θ*,*i*_	Gamma (5.0, 20.0)	Gamma (*μ*_*θ*,*i*_, 500.0)
$\sigma_{\theta ,i}^{2}$	Gamma (0.01*μ_*θ*,*i*_, 1.5)	Gamma ($\sigma_{\theta ,i}^{2}$, 1.5)
*ρ*_*θ*_	Uniform (-0.1, 0.1)	Uniform (-0.1, 0.1)
*α*_*i*_	Uniform (0.0, 2.0)^1^	Uniform (0.0, 2.0)
$h_{i}^{2}$	Uniform (0.2, 0.8)	Uniform ($h_{i}^{2}-0.1,\;h_{i}^{2}+0.1$)
*V*_*i*_	1.0	1.0
*m*_*i*_	Gamma (0.001, 10.0)	Gamma (*m*_*i*_, 100.0)
*n*_*i*_	Gamma (1000.0, 10.0)	Gamma (*n*_*i*_, 100.0)

^1^ When simulating data, *α*_*i*_ was drawn from this distribution only after first clearing a hurdle value of 0.25. Failure to clear the hurdle resulted in *α*_*i*_ being equal to zero.

## Results

### Evaluating performance using simulated data

To evaluate the performance of our approach, we applied it to a large number of simulated data sets. Specifically, we drew the parameters described in Table 1 at random and simulated evolution within metapopulations consisting of 5, 10, and 20 populations. Each simulation assumed migration followed an island model and continued until the metapopulation reached an approximate equilibrium where the statistical moments describing the multivariate distribution of population mean phenotypes remained approximately constant over time. If an equilibrium was not reached within 500 generations, the simulation was halted and parameters drawn again at random. At the completion of each simulation, the summary statistics, *μ*_*x*_, *μ*_*y*_, *σ*_*x*_, *σ*_*y*_, and *ρ*_*xy*_ were recorded. Once data had been simulated, we used our ABC method to develop posterior distributions for the parameters in Table 1, focusing our assessment of accuracy on the coevolutionary sensitivity in each species, *α*_*i*_, and a composite index for the strength of coevolution equal to $\sqrt{\alpha_{x}\alpha_{y}}$. We studied the performance of our method for two different scenarios. In the first, we assumed little independent biological information was available to inform prior distributions of background parameters (e.g., rates of gene flow, effective population sizes, etc.) such that prior distributions for these parameters were broad and restricted only by biological plausibility. In the second, we assumed independent biological information (e.g., molecular studies, experiments, etc.) was available and could be used to refine prior distributions for background parameters.

*Unrefined priors–*We first considered the power and performance of our method when applied to a biological system where only the trait means of the interacting species are known across populations. In such situations, prior distributions for the background parameters required by our method are constrained only by biological intuition and plausibility. Consequently, the modes of prior distributions could be very far from the actual parameters used to generate simulated data. To evaluate performance under this worst-case scenario, prior distributions were assumed to be identical to the distributions from which parameters used to simulate data were drawn with two exceptions. First, prior distributions for parameters defining the strength of stabilizing selection (*γ*_*x*_, *γ*_*y*_) were assumed to follow uniform distributions informed by meta-analyses [50, 51]. Second, distributions from which the coevolution parameters were drawn for simulation differed from the prior distributions by including a “hurdle”. Specifically, the simulations drew the coevolution parameters from uniform hurdle distributions that enriched the probability of drawing parameters uniquely equal to zero. Using hurdle distributions allowed us to calculate Type I error rates for our method by guaranteeing “control” simulations were performed where biotic selection was absent for one or both of the species. A detailed description of the distributions used to draw parameters for the simulations and the prior distributions can be found in Table 2.

For each simulated data set, the ABC method was run until 200 points were in the posterior distribution. Although 200 points is far too few to achieve a reliable estimate for any individual simulated data set, it allowed us to explore a much greater diversity of simulated data sets in a reasonable amount of time. Because so few points were included in the posterior, it is likely our results represent the worst-case scenario for the performance of our method. Later, when we apply our method to real data, we vastly increase the number of points in the posterior. Acceptance into the posterior distribution required that the spatial averages of population mean phenotypes be within 0.1+15% of their observed values, that the spatial standard deviations of population mean phenotypes be within 0.1+20% of their observed values, and that the spatial correlation between population mean phenotypes be within 25% of its observed value. These thresholds were chosen to balance the competing demands of acceptance rate and accuracy in a way that allowed us to explore the performance of our method over a large number of simulated data sets. For each simulated data set, we calculated the estimated values for the coevolutionary sensitivities, *α*_*i*_, by identifying the modes of their marginal posterior distributions. We also calculated a composite strength of coevolution, $\sqrt{\alpha_{x}\alpha_{y}}$, that integrates the coevolutionary sensitives of each species into a single numeric score. Ninety five percent credible intervals were calculated for these quantities as the interval of highest posterior density (HPD) in the marginal posterior distribution. Although relying on the marginal distributions for these key parameters (rather than the full multivariate distribution) could, in principle, be problematic, initial simulations suggested the posterior distributions for these key parameters are approximately independent in most cases. Focusing on only the marginal distributions allowed us to get more reliable estimates with fewer points in the posterior, and thus allowed us to study a larger number of simulated data sets.

**Fig 1:**
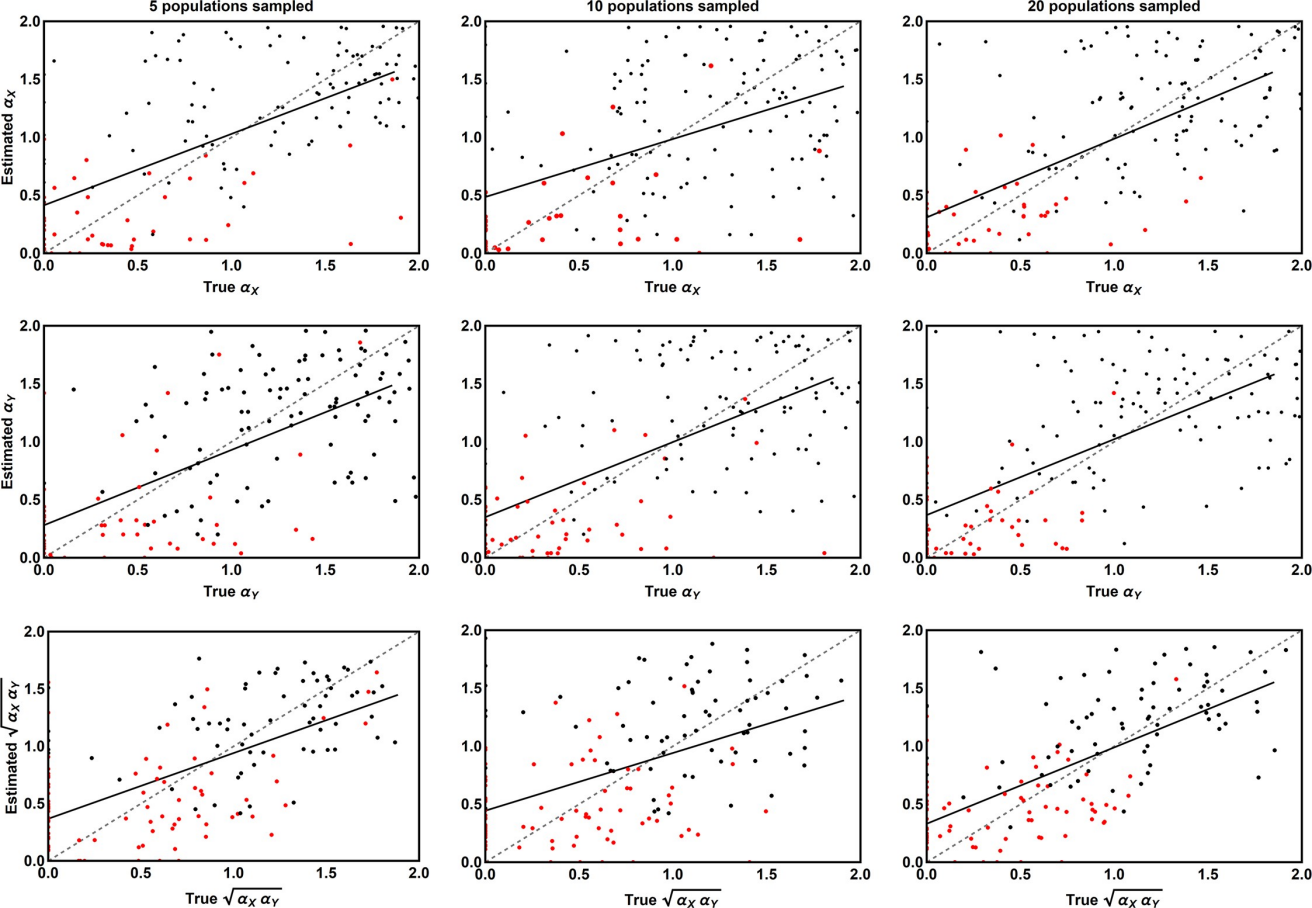
The relationship between the coevolutionary sensitivities used to simulate data (X axis) and the values of these parameters estimated by our ABC method (Y axis) for scenarios where independent estimates of background parameters are unavailable, necessitating the use of relatively broad prior distributions. The left-hand column shows results for cases where only 5 populations have been sampled, the center column cases where 10 populations have been sampled, and the right column cases where 20 populations have been sampled. Red points indicate parameter estimates for which the associated credible interval did not include zero. Black points indicate parameter estimates with credible intervals overlapping zero. The solid black line is the best linear fit and the dashed gray line is the perfect one to one relationship expected if all estimates were equal to their true values.

**Fig 2:**
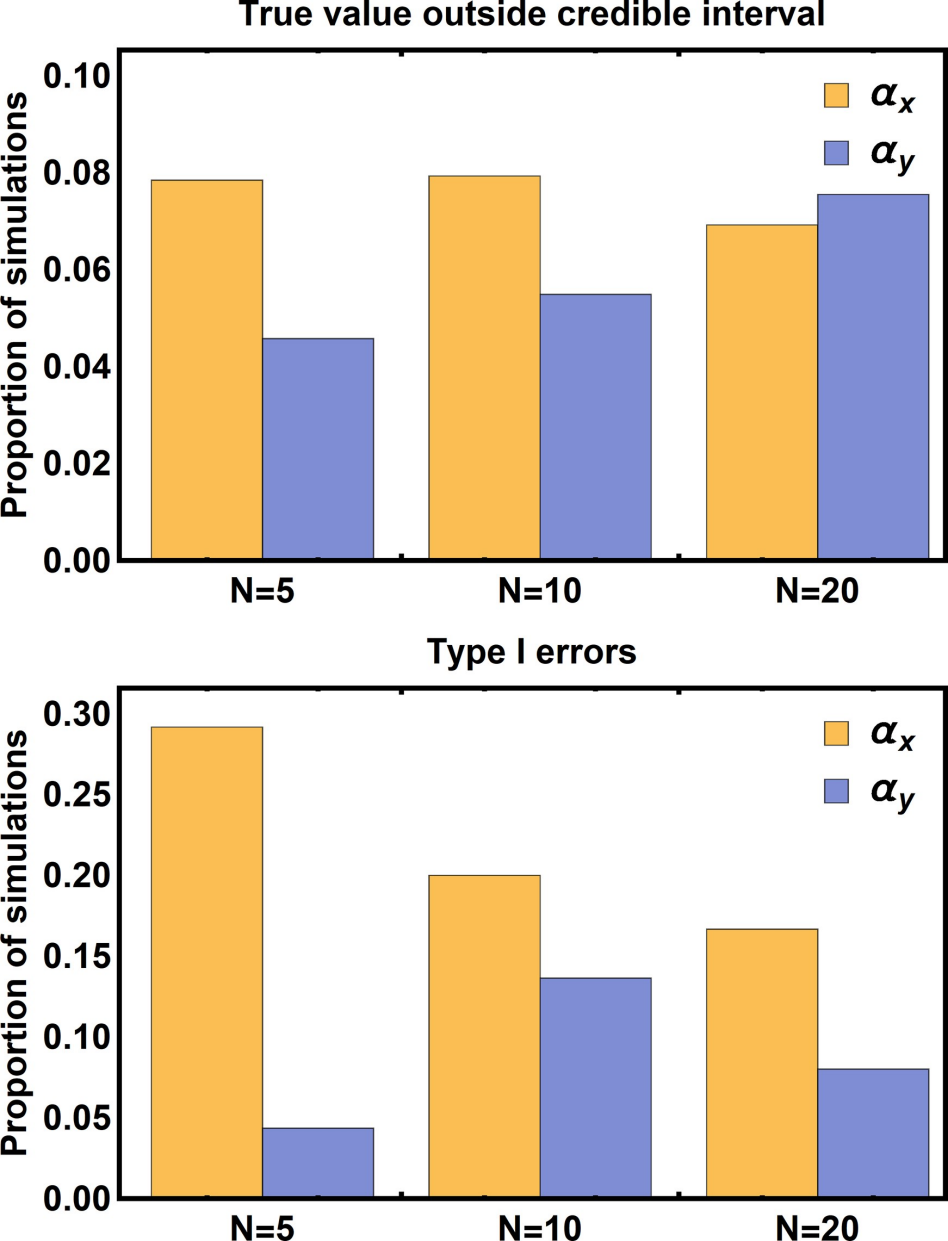
The proportion of simulations where the true value of the parameter fell outside of the 95% credible interval of its posterior distribution (top panel) and the proportion of simulations where the true value of the parameter was equal to zero, but the credible interval did not include zero (bottom panel). Inference was performed using very broad priors restricted only by biological feasibility.

We applied our ABC method to 155 simulated data sets where 5 populations were sampled, 166 simulated data sets where 10 populations were sampled, and 162 simulated data sets where 20 populations were sampled. Performance was evaluated in two ways. First, we compared the true values of the coevolutionary sensitivities to their values estimated by the modes of their marginal posterior distributions (Fig 1). This comparison revealed that our method did a reasonable job of estimating the coevolutionary sensitivities for each species, and the composite strength of coevolutionary selection (Fig 1). Next, we calculated the percentage of cases in which the true values of the coevolutionary sensitivities fell outside their 95% credible intervals (Fig 2). This demonstrated that the error rates of our estimates were slightly inflated, with between 4%-8% of estimates lying outside the 95% credible interval (Fig 2). Similarly, analysis of Type I error rates demonstrated positive values of the coevolutionary sensitivities were erroneously inferred in between 4%-30% of cases, although when twenty populations were sampled the Type I error rates fall to more reasonable values between 8%-17%.

**Fig 3:**
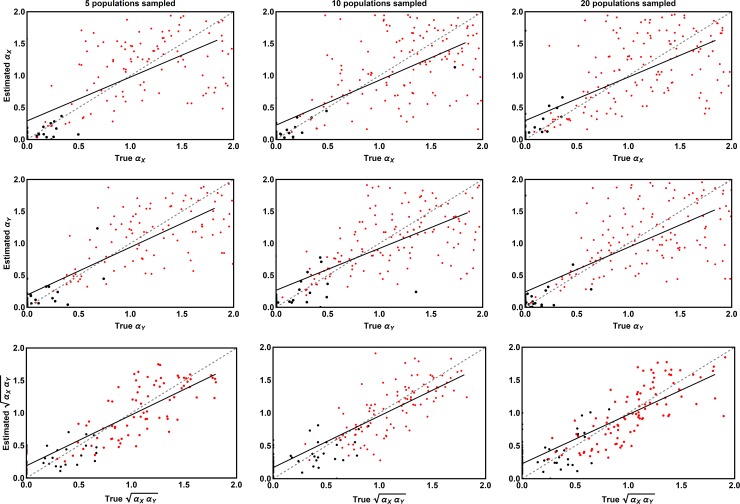
The relationship between the coevolutionary sensitivities used to simulate data (X axis) and the values of these parameters estimated by our ABC method (Y axis) for scenarios where prior distributions for background parameters have been refined using independent estimates. The left-hand column shows results for cases where only 5 populations have been sampled, the center column cases where 10 populations have been sampled, and the right column cases where 20 populations have been sampled. Red points indicate parameter estimates for which the associated credible interval did not include zero. Black points indicate parameter estimates with credible intervals overlapping zero. The solid black line is the best linear fit and the dashed gray line is the perfect one to one relationship expected if all estimates were equal to their true values.

**Fig 4:**
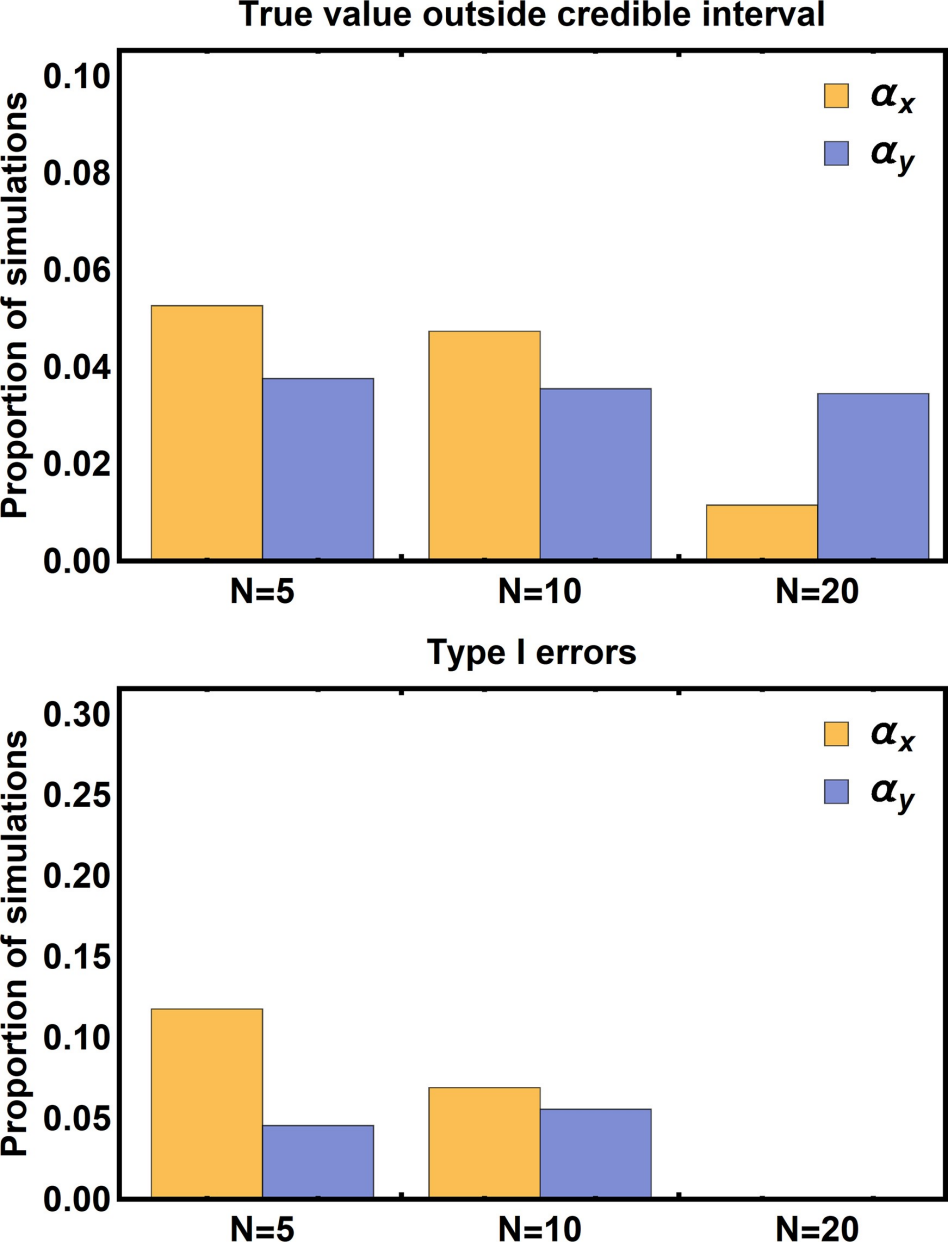
The proportion of simulations where the true value of the parameter fell outside of the 95% credible interval of its posterior distribution (top panel) and the proportion of simulations where the true value of the parameter was equal to zero, but the credible interval did not include zero (bottom panel). Inference was performed assuming independent estimates for background parameters were available, allowing narrower prior distributions to be used than in Fig 2.

*Refined priors–*In some cases, such as the Camellia-Weevil interaction we will apply our method to in the next section, independent estimates for background parameters are available, allowing for increased refinement of prior distributions. We studied such scenarios by centering the prior distributions for the background parameters on the values used to generate the simulated data (Table 3). This analysis proceeded identically to that described in the previous section except that the method was tested using 136 simulated data sets with 5 populations sampled, 172 simulated data sets with 10 populations sampled, and 177 simulated data sets with 20 populations sampled. Not surprisingly, when background parameters have been estimated independently and accurately, the performance of our method is substantially improved (Figs 3 and 4). Specifically, sampling from ten or more populations now guarantees the true values of the coevolutionary sensitivities reside within their 95% credible intervals in at least 95% of simulations as desired (Fig 4). Similarly, as long as ten or more populations are sampled, the Type I error rates for the coevolutionary sensitivities remain below 5% (Fig 4).

### Application: Armament escalation between plant and seed predator

The interaction between the Japanese camellia, *Camellia japonica*, and its obligate seed predator, *Curculio camelliae*, is a textbook example of a coevolutionary arms race [34]. Japanese camellias defend against weevil attack using a thickened pericarp that defends the seeds inside from the weevil’s attempts to drill through the defensive pericarp using its elongated rostrum. Key features of this interaction include striking exaggeration of the traits mediating the interaction (rostrum length in the weevil and pericarp thickness in the camellia) and a strong statistical association between plant and weevil traits over the geographic range of the interaction. Through a lengthy series of field studies, elegant experiments, and genetic work, coevolution has been established as the most likely explanation for these unusual features of the interaction [32, 52–55]. Despite the extensive research effort devoted to this system, however, we lack quantitative estimates for the strength of coevolution, other than those we have recently derived using maximum likelihood (Week and Nuismer, 2019). In this section, we apply our ABC method to this system, capitalizing on the extensive body of existing research to define prior distributions for key background parameters.

Trait data–The phenotypic data we analyze comes from studies of this interaction that estimated population mean pericarp thickness and population mean rostrum lengths across 17 populations in Japan [34]. Because our method assumes an island model of migration, we restricted our analysis to the subset of these populations that formed a single cluster within population genetic analyses [56]. This resulted in a final data set consisting of estimates for population mean phenotypes in 13 populations (S1 Table).

Prior distributions–Previous research in this system provides a solid grounding for prior distributions of most background parameters. For instance, effective population sizes (*n*_*i*_) and rates of gene flow (*m*_*i*_) have been estimated for both camellia and weevil [54, 55]. Heritability ($h_{i}^{2}$) has been estimated for pericarp thickness directly [53] and can be at least crudely guessed and bounded for rostrum length using estimates for related species [54]. The phenotypic optima favored by stabilizing selection (*μ*_*θ*,*i*_) can also be estimated independently from previous work. Specifically, the optimum trait value for the weevil can be at least crudely estimated using rostrum lengths of male weevils, because male weevils do not use their rostra in interactions with the plant [53]. Thus, as long as male and female rostrum lengths are not genetically correlated (or the population is at equilibrium), male rostrum length should serve as a reasonable proxy for the optimum rostrum length in the absence of interaction with the Camellia. Unfortunately, the optimum trait value for the camellia must be estimated using populations outside the range of the weevil, and such estimates could easily be confounded by spatial variation [53]. Consequently, we use rather broad parameters for these parameters to capture this uncertainty. Similarly, the spatial variance in these phenotypic optima can be crudely estimated as the variance in rostrum length in male weevils from different populations and the variance in pericarp thickness from different populations outside the range of the weevil [53]. Phenotypic variance (*V*_*i*_) can be estimated for each species by averaging the within population phenotypic variance for each trait over all thirteen populations included in our analysis. Unfortunately, the strengths of stabilizing selection (*γ*_*i*_) have not been independently estimated, forcing us to rely on broad priors for these parameters informed only by previous meta-analyses of the strength of stabilizing selection across studies and taxonomic groups [50, 51]. Prior distributions for all model parameters are described in Table 4.

**Table 4:** Prior distributions for camellia and weevil parameters.

*Model Parameter*	*Weevil Distribution*	*Camellia Distribution*
*γ*_*i*_	Gamma (0.05, 2.0)	Gamma (0.05, 2.0)
*μ*_*θ*,*i*_	Gamma (5.912, 8.0)	Gamma (6.248, 8.0)
$\sigma_{\theta ,i}^{2}$	Gamma (0.15, 1.05)	Gamma (1.27, 1.3)
*ρ*_*θ*_	Uniform (-0.1, 0.1)	
*α*_*i*_	Uniform (0.0, 3.0)	Uniform (0.0, 3.0)
$h_{i}^{2}$	Uniform (0.312, 0.512)	Uniform (0.6375, 0.8375)
*V*_*i*_	Gamma (0.71, 50.0)	Gamma (3.72, 50.0)
*m*_*i*_	Gamma (3.07×10^−6^, 2.0)	Gamma (1.00×10^−6^, 2.0)
*n*_*i*_	Gamma (30870, 200.0)	Gamma (1818, 100.0)

**Fig 5:**
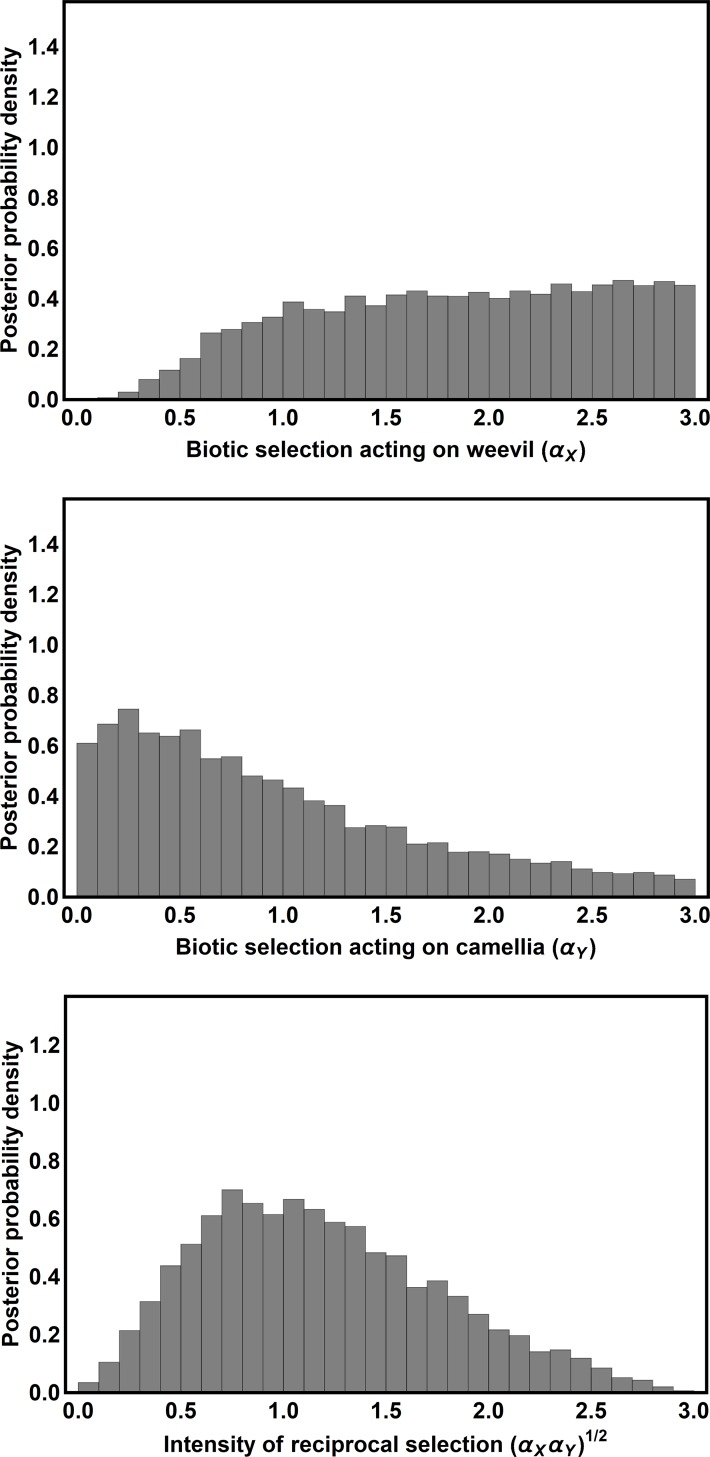
Posterior probability densities for weevil coevolutionary sensitivity (top panel), camellia coevolutionary sensitivity (middle panel), and the composite index of reciprocal selection (bottom panel). Modal values were 2.37, 0.21, 0.81 respectively, and 95% credible intervals were {0.60, 2.94}, {0, 2.40}, {0.15, 2.20} respectively.

Posterior distributions and coevolutionary inference–The ABC algorithm was run until there were 7,513 points in the posterior distribution, using acceptance thresholds somewhat more restrictive than those used in method performance evaluations. Specifically, parameter combinations were passed to the posterior distribution only when the spatial average population mean phenotypes were within 1.120mm and 0.887mm of their values in the empirical data for weevil and camellia, respectively, the standard deviations among population mean phenotypes were within 0.327mm and 0.553mm of their values for weevil and camellia, and the correlation between weevil and camellia mean phenotypes was within 0.224 of its value in the data. The modal values of the coevolutionary sensitivities were then identified, as were their 95% credible intervals (HPD). Posterior distributions are reported in Fig 5. The results demonstrate that the mode for weevil coevolutionary sensitivity (*α*_*W*_) is equal to 2.37 with a 95% credible interval between 0.60 and 2.94. Thus, our results support the idea that Camellia pericarp thickness exerts selection on weevil rostrum length. For the Camellia, our results demonstrate the mode for Camellia coevolutionary sensitivity (*α*_*C*_) is equal to 0.21 with a 95% credible interval between 0 and 2.40. Thus, our results are consistent with the hypothesis that weevil rostrum length exerts selection on Camellia pericarp thickness, but cannot rule out the possibility that pericarp thickness evolves independently of weevil rostrum length. In summary, the results of our ABC analysis point to reciprocal selection and coevolution as the most likely scenario, but do not preclude the possibility that evolution is unilateral, with weevil rostrum length tracking independently evolving Camellia pericarp thickness.

**Fig 6:**
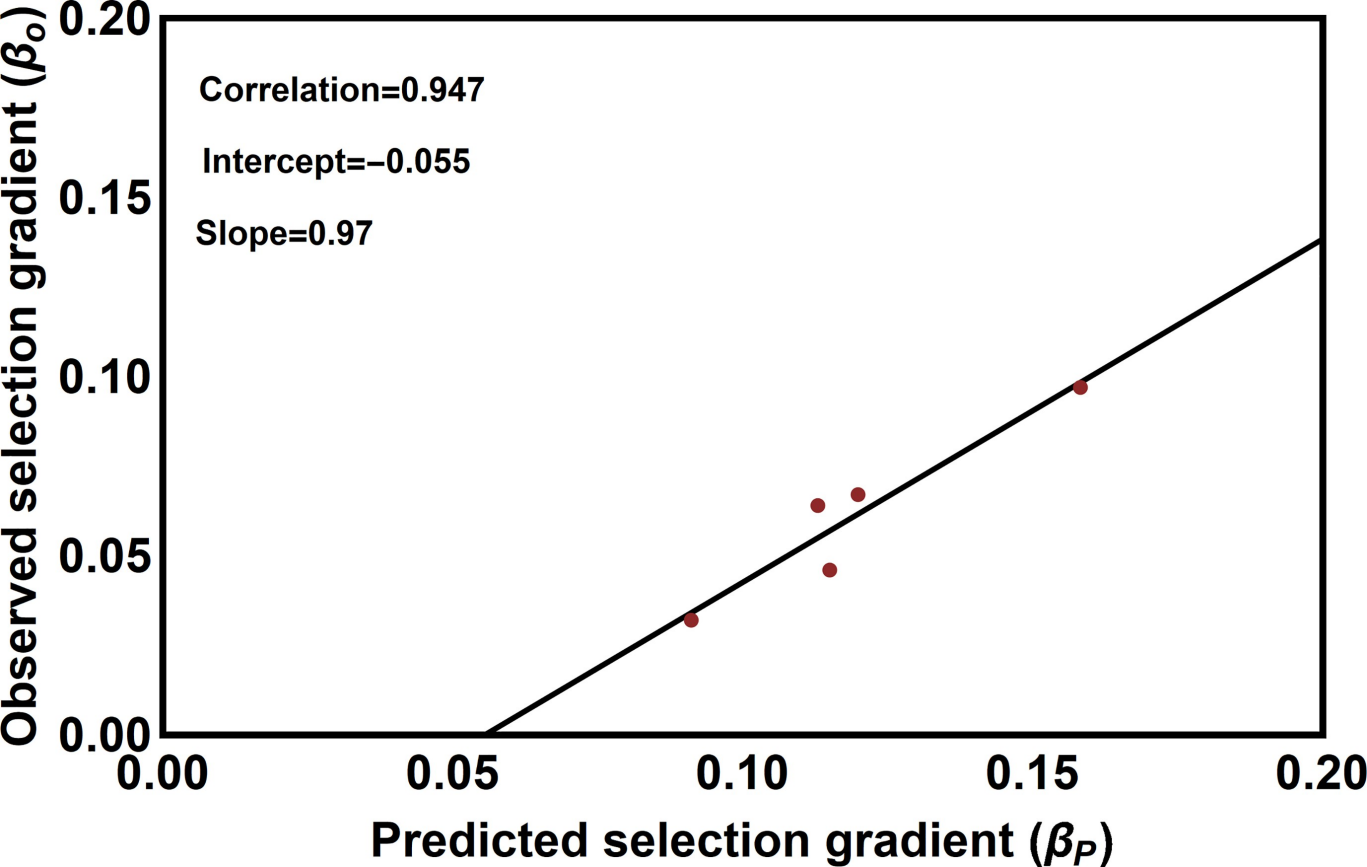
Standardized biotic selection gradients acting on pericarp thickness predicted by our ABC method plotted against their independently measured values. Predicted selection gradients were calculated for the five populations for which Toju and Sota [34] reported significant selection gradients and which belonged to a single clade. Predicted selection gradients were calculated by conducting a simulated phenotypic selection analysis in each population based on the modal parameter values of the posterior distributions.

Validation against independent estimates of selection– Although estimates of population mean phenotypes are not available from enough populations for standard methods of cross-validation to be useful [e.g., 57], previous work estimating selection gradients acting on pericarp thickness within a number of populations [34] allows independent validation of our estimates for the coevolutionary sensitivities. Specifically, using the coevolutionary sensitivities estimated by our ABC method, we can predict the standardized biotic selection gradient for any population where trait means are known. Applying this approach to the five populations for which significant selection gradients acting on pericarp thickness have been previously reported [34] resulted in a correlation of 0.97 between predicted and observed values (Fig 6). Although strongly correlated, our simulated selection gradients consistently overestimated values measured directly from the data. This discrepancy may arise because our mathematical model fails to capture some of the nuanced functional relationship between weevil rostrum length and camellia pericarp thickness. It is also possible, however, that this apparent discrepancy is nothing more than noise stemming from the small sample sizes used in the empirical study and the small number of populations for which significant estimates of selection are available. Resolving this apparent discrepancy will require data from a larger number of populations and exploration of alternative mathematical formulations. As a whole, however, we take the general agreement between our predicted selection gradients and those directly and independently estimated through phenotypic selection analysis as support for the validity of our approach.

## Discussion

We have developed an approximate Bayesian methodology (*ABC Coevolution*) for estimating the strength of coevolutionary selection using the spatial distribution of trait means. Our approach relaxes key assumptions of an existing maximum likelihood technique and performs reliably when population mean trait values are sampled from ten or more populations and independent information is available to refine prior distributions for background parameters. Specifically, when priors are broad and informed only by biological plausibility, the true values of the coevolutionary sensitivities lie outside of their 95% credible intervals in up to 8% of simulated data sets (Fig 2). In contrast, when independent estimates of background parameters can be used to refine priors, the true values of the coevolutionary sensitivities lie outside their 95% credible interval in fewer than 5% of cases, as long as 10 or more populations are sampled (Fig 4). Applying our method to the well-studied interaction between the plant, *Camellia japonica*, and its seed predator, *Curculio camelliae*, provides support for the hypothesis of a coevolutionary arms race between armament and defense, but fails to unequivocally rule out the possibility of unilateral trait escalation.

Comparing the estimates for coevolutionary selection in the *C*. *camellia–C*. *japonica* derived here with those we previously derived using a maximum likelihood approach (Week and Nuismer, 2019) reveals qualitative similarity (i.e., coevolution is the best supported hypothesis) but quantitative discrepancy. Specifically, the estimates of coevolutionary selection we derive here are much larger than those inferred using maximum likelihood, even after transforming the previous estimates to the same scale of measurement. There are at least three reasons the ABC approach infers a greater magnitude of coevolutionary selection than the maximum likelihood approach. First, the two approaches assume different functional forms of interaction between the species. Second, the maximum likelihood approach assumes only random genetic drift generates spatial variation in trait means. Because drift is a weak force in all but the smallest of populations, spatial variation can be maintained only when stabilizing selection and coevolutionary selection are also very weak. If this were not the case, stabilizing and coevolutionary selection would overwhelm drift and erode spatial variation. By allowing the optimal trait values favored by stabilizing selection to vary across space, the ABC approach avoids this trap and can maintain spatial variation even when stabilizing and coevolutionary selection become strong. Third, the maximum likelihood approach assumes the outcome of interactions does not depend too strongly on the traits of the interacting individuals, allowing analytical approximations for evolutionary change to be derived. Although mathematically convenient, this assumption guarantees the maximum likelihood approach will underestimate the true magnitude of coevolutionary selection. In contrast, the ABC approach developed here avoids this assumption by relying on brute force simulation and so can return estimates of coevolutionary selection that are much greater in magnitude. In short, the maximum likelihood approach is faster and more computationally efficient but will underestimate the strength of coevolutionary selection in cases where its true value is strong.

Although the Bayesian approach we develop here relaxes several important assumptions of our earlier maximum likelihood approach (e.g., weak coevolutionary selection, absence of gene flow, spatially homogenous abiotic optima), it still makes important assumptions that may not be satisfied in all systems. For instance, as currently implemented, our approach does not allow the strength of coevolutionary selection to vary over space, and thus ignores the potential for selection mosaics [58–60]. An obvious, and relatively straightforward, extension of the Bayesian methodology developed here would include such selection mosaics. However, initial explorations of this possibility suggested accurate inference will require sampling trait means from many more populations than what is generally available, even in very well-studied interactions like those between *C*. *camellia* and *C*. *japonica*. In addition, just as with our previous likelihood-based method, the approach developed here assumes the metapopulation has reached an evolutionary equilibrium, at least with respect to the statistical moments we use as summary statistics. In cases where time series information on traits is available, or it is possible to establish times of divergence, developing non-equilibrium approaches may offer promising alternatives. Our approach also relies upon the temporal constancy of key parameters such as heritabilities, phenotypic variances, abiotic optima, and strengths of stabilizing and coevolutionary selection. Although allowing these parameters to vary over time is relatively straightforward from a programming/computational standpoint, doing so seems wildly premature given we lack sufficient data to establish even ballpark priors for how these parameters change over time in natural systems. Finally, we focus here on the special case of an island model where gene flow occurs equally among all populations. Extending our approach to cases that generate isolation by distance, such as stepping stone models, will allow application to a broader range of biological systems.

In summary, we have presented a novel Bayesian methodology for estimating the strength of coevolutionary selection driving putative arms races between pairs of interacting species. Although we have restricted our attention to arms races between species, adapting our approach to arms races within species, such as putative cases of runaway sexual selection or conflict between sexes or groups within a species [e.g., 15, 61, 62], is a straightforward matter. Similarly, adapting our approach to other forms of ecological interactions such as mutualism or competition or to other mechanisms such as phenotype matching, is extremely straightforward and requires only minor modifications to the source code. Implementing these and other options in our inference package (*ABC coevolution*) will be a central goal of future development. Broad application of the approach developed here provides an opportunity to better understand the distribution of coevolutionary selection across interactions, communities, and ecosystems, and to answer long-standing debates such as the importance of reciprocity in the evolutionary process [63, 64].

## Supporting information

S1 Table Trait data for Camellia japonica and *Curculio camelliae*. Taken from Table 1 in Toju and Sota (2006a).
(DOCX)
