# Supplementary material for: Approximate Bayesian estimation of coevolutionary arms races
Source: PLoS Comput Biol. 2019 Apr 15;15(4):e1006988. doi: 10.1371/journal.pcbi.1006988 (PMC6483265; doi:10.1371/journal.pcbi.1006988)
Supplement: S1 Table — Taken from Table 1 in Toju and Sota (2006a). (DOCX) [file pcbi.1006988.s001.docx]

S1 Table. *Camellia japonica* and *Curculio camelliae* data. Taken from Table 1 in Toju and Sota (2006a).

| Locality | Mean rostrum length (mm) | Mean pericarp thickness (mm) |
| --- | --- | --- |
| Kutsuki | 10.42 | 6.07 |
| Kyoto | 9.89 | 6.3 |
| Jurinji | 10.31 | 6.13 |
| Nara | 10.05 | 6.66 |
| Kiikatsuura | 10.66 | 7.73 |
| Taiji | 9.12 | 6.76 |
| Arafune | 9.21 | 6.42 |
| Kiioshima | 9.61 | 7.52 |
| Muroto | 10.06 | 7.77 |
| Usa | 13.63 | 11.65 |
| Ashizuri | 12.98 | 12.8 |
| Reihoku | 11.68 | 11.89 |
| Takahama | 11.48 | 11.13 |
